# Sulforaphane Inhibits Oxidative Stress and May Exert Anti-Pyroptotic Effects by Modulating NRF2/NLRP3 Signaling Pathway in *Mycobacterium tuberculosis*-Infected Macrophages

**DOI:** 10.3390/microorganisms12061191

**Published:** 2024-06-13

**Authors:** Guangxin Chen, Lin Shen, Hong Hu, Yazhi Feng, Da Wen, Yiyao Liu, Huizhe Zhai, Wei Sun, Meifen Wang, Xinghua Lei, Ping Li, Qiuhong Xiong, Changxin Wu

**Affiliations:** 1 Institutes of Biomedical Sciences, Shanxi University, Taiyuan 030006, China; 19581550438@163.com (L.S.); huhong0121@163.com (H.H.); fyz_99@163.com (Y.F.); wdbio@outlook.com (D.W.); kellyliu0914@163.com (Y.L.); 15253381553@163.com (H.Z.); 202223119019@email.sxu.edu.cn (W.S.); mmeiffwanggg@163.com (M.W.); lxh072709@163.com (X.L.); pingli@sxu.edu.cn (P.L.); qxiong@sxu.edu.cn (Q.X.); 2 Shanxi Provincial Key Laboratory of Medical Molecular Cell Biology, Taiyuan 030006, China; 3 Shanxi Provincial Key Laboratory for Prevention and Treatment of Major Infectious Diseases, Taiyuan 030006, China

**Keywords:** sulforaphane, macrophages, oxidative stress, pyroptosis

## Abstract

Sulforaphane (SFN) is a natural isothiocyanate derived from cruciferous vegetables such as broccoli, Brussels sprouts, and cabbage. SFN plays a crucial role in maintaining redox homeostasis by interacting with the active cysteine residues of Keap1, leading to the dissociation and activation of NRF2 in various diseases. In this study, our objective was to investigate the impact of SFN on oxidative stress and pyroptosis in *Mycobacterium tuberculosis* (*Mtb*)-infected macrophages. Our findings demonstrated that *Mtb* infection significantly increased the production of iNOS and ROS, indicating the induction of oxidative stress in macrophages. However, treatment with SFN effectively suppressed the expression of iNOS and COX-2 and reduced MDA and ROS levels, while enhancing GSH content as well as upregulating NRF2, HO-1, and NQO-1 expression in *Mtb*-infected RAW264.7 macrophages and primary peritoneal macrophages from WT mice. These results suggest that SFN mitigates oxidative stress by activating the NRF2 signaling pathway in *Mtb*-infected macrophages. Furthermore, excessive ROS production activates the NLRP3 signaling pathway, thereby promoting pyroptosis onset. Further investigations revealed that SFN effectively suppressed the expression of NLRP3, Caspase-1, and GSDMD, IL-1β, and IL-18 levels, as well as the production of LDH, suggesting that it may exhibit anti-pyroptotic effects through activation of the NRF2 signaling pathway and reductions in ROS production during *Mtb* infection. Moreover, we observed that SFN also inhibited the expression of NLRP3, ASC, Caspase1, and IL-1β along with LDH production in *Mtb*-infected primary peritoneal macrophages from NFR2^−/−^ mice. This indicates that SFN can directly suppress NLRP3 activation and possibly inhibit pyroptosis initiation in an NRF2-independent manner. In summary, our findings demonstrate that SFN exerts its inhibitory effects on oxidative stress by activating the NRF2 signaling pathway in *Mtb*-infected macrophages, while it may simultaneously exert anti-pyroptotic properties through both NRF2-dependent and independent mechanisms targeting the NLRP3 signaling pathway.

## 1. Introduction

Tuberculosis (TB), a severe infectious disease, has afflicted humanity for centuries. TB is caused by infection with *Mycobacterium tuberculosis* (*Mtb*) and primarily affects the pulmonary tissue. However, it can also impact the abdomen, genitourinary tract, gastrointestinal tract, skin, bones, nervous system, and other lymph node-containing organs, known as extrapulmonary TB [1,2]. TB stands as the second leading cause of mortality attributed to a single infectious agent worldwide, only surpassed by COVID-19 inducing severe acute respiratory syndrome [3]. Over 10 million individuals contract TB annually, and an estimated quarter of the global population carries this infection [4]. The mortality rate among untreated TB patients reaches approximately 50% [5]. Treatments recommended by the World Health Organization (WHO) involve the oral administration of anti-TB drugs for a duration of 4 to 6 months. The emergence of rifampicin-resistant TB (RR-TB) and multidrug-resistant TB (MDR-TB) has heightened challenges in managing this disease. Consequently, urgent measures such as screening potential anti-TB drugs, developing more efficacious vaccines, and identifying prospective therapeutic targets should be prioritized.

The interaction between macrophages and *Mtb* is pivotal in the pathogenesis of TB [6]. Upon invasion into the human body, *Mtb* is recognized and engulfed by phagocytic cells such as macrophages and dendritic cells [7], and NADPH oxidase 2 receptors (NOX2) are activated to facilitate the generation of reactive oxygen species (ROS), including reactive oxygen intermediates (ROIs), active nitrogen intermediates (RNIs), and pro-inflammatory cytokines [8,9]. ROS can directly eliminate pathogens by inducing oxidative damage to biological components such as DNA and proteins while also serving as signaling molecules for downstream non-oxidative mechanisms. However, prolonged infection leads to excessive ROS levels, causing oxidative stress that exacerbates TB progression. Moreover, ROS can activate NLRP3 as a signaling molecule, triggering the pyroptosis of *Mtb*-infected macrophages. The death of host cells promotes the release and dissemination of *Mtb*, which hampers effective control. Therefore, targeting oxidative stress and pyroptosis may represent promising therapeutic strategies for TB.

SFN is a natural glucosinolate belonging to the isothiocyanate family, which can be found in seeds and mature plants, particularly in sprouts of cruciferous vegetables such as cabbage, broccoli, cauliflower, and Brussels sprouts [10]. Due to its lipophilicity and molecular size, SFN easily permeates the cell membrane and diffuses into the cytoplasm [11,12], exhibiting multiple functionalities. SFN has demonstrated preventive or therapeutic effects against various tumors and cancers including pancreatic cancer [13], leukemia [14], prostate cancer [15], liver cancer [16], lymphoma [17], and bladder carcinoma [18]. Moreover, SFN also exhibits neuroprotective properties against neurodegenerative diseases like Alzheimer’s disease [19], Parkinson’s disease [20,21], and multiple sclerosis [22]. Additionally observed are potential anti-obesity effects of SFN [23,24]. The health-promoting effects of SFN primarily stem from its antioxidant activity, anti-inflammatory properties, as well as its ability to inhibit apoptosis. Amongst its diverse biological activities lies an exceptional role for SFN as a pivotal regulator of redox homeostasis while exerting cytoprotective effects through the activation of the transcription nuclear factor erythroid 2-related factor (NRF2).

NRF2, a member of the Cap-n-Collar family of basic leucine zipper proteins, is widely recognized as the primary cellular sensor of oxidative stress [25]. Under normal conditions, NRF2 is sequestered by cytoplasmic Keap1 and targeted for proteasomal degradation [26]. However, in the presence of oxidative stress, the interaction between NRF2 and Keap1 gradually dissociates, allowing free and newly synthesized NRF2 to translocate into the nucleus and form heterodimers with small Maf proteins. NRF2 regulates approximately 500 genes encoding proteins involved in maintaining redox balance, detoxification processes, stress response mechanisms, and metabolic enzyme activity [27]. Therefore, the activation of NRF2 may play a pivotal role in regulating oxidative stress during *Mtb* infection. The aim of this study is to investigate the effects of SFN on oxidative stress and pyroptosis in macrophages infected with *Mtb*.

## 2. Materials and Methods

### 2.1. Mtb Culture

The *Mtb* (H37Ra) was purchased from Gene Optimal Biotechnology Co., Ltd. (Shanghai, China) and cultured in a 7H9 complete culture which contained 10% OADC (Middlebrook, NY, USA) at 30 °C.

### 2.2. Cell Culture

RAW264.7 macrophages were provided by Hycyte Biotechnology Co., Ltd. (Quanzhou, China). Cells were cultured in DMEM (Gibco, Grand Island, NY, USA) with 10% FBS (Sorfa, Beijing, China) in a humidified chamber (37 °C, 5% CO_2_).

### 2.3. Primary Peritoneal Macrophages Isolation

The peritoneal cavity of WT and NRF2^−/−^ mice was intraperitoneally injected with 3 mL of 3% thioglycollate broth for a duration of 3 d. Subsequently, all mice were sacrificed, and the peritoneal cavity was flushed with 4 mL of cold PBS while gently kneading the abdomen for 5 min. The collected fluid containing primary macrophages from the peritoneal cavity was then centrifuged at 1000 rpm for 5 min. RPMI1640 medium supplemented with 10% FBS was utilized to resuspend and seed the cells in a culture dish. After a period of 2 h, nonadherent cells were discarded, leaving behind primary macrophages as adherent cells.

### 2.4. Western Blot Assay

Cells were lysed using RIPA buffer (Solarbio, Beijing, China), and the supernatants were concentrated using the Pierce BCA protein assay kit (Thermo, Rockford, IL, USA). Protein samples were separated by electrophoresis on SDS-PAGE gels at 110 V for 1.5 h. Subsequently, the proteins were transferred to PVDF membranes by electroblotting at 110 V for 1 h. The PVDF membranes containing protein were blocked with 5% nonfat milk in TBS-T at room temperature for 2 h. After washing with TBS-T, the PVDF membranes were incubated with primary antibodies at 4 °C overnight or at room temperature for 1 h. Following another round of washing with TBS-T, HRP-conjugated secondary antibodies in TBS-T solution were used to incubate the PVDF membranes at room temperature for 1 h. Blots were developed using supper ECL (CWBIO, Beijing, China). The primary antibodies were iNOS (CST, Danvers, MA, USA), COX-2, NRF2, HO-1, NQO-1, β-actin, HRP-conjugated secondary antibody (ABclonal, Wuhan, China), NLRP3, ASC, Caspase-1, GSDMD, and IL-1β (Bioss, Beijing, China).

### 2.5. Cellular Immunofluorescence Assay

After infection, the cells were washed three times with cold PBS (each for 5 min). Subsequently, the cells were fixed with 4% paraformaldehyde for 10 min and then washed again with cold PBS. Following this, the cells were treated with 0.1% Triton X-100 in PBS for 10 min and underwent another round of washing with cold PBS. Incubation of the cells in a solution of 5% BSA dissolved in PBS was carried out for 1 h at room temperature. Next, the cells were incubated overnight at 4 °C with anti-NRF2 primary antibody (diluted to a ratio of 1:300) in PBS containing 5% BSA, followed by washing once more with cold PBS. This was succeeded by incubating the cells at room temperature for an additional 1 h using Donkey anti-Rabbit IgG (H+L) Highly Cross-Adsorbed Secondary Antibody, Alexa Fluor™488 (diluted to a ratio of 1:2000), and subsequent washing again with cold PBS. Finally, DAPI was used to incubate the cells for a duration of 5 min before undergoing another round of washing using cold PBS. Images were observed and recorded utilizing a Nikon fluorescence microscope (Nikon, Tokyo, Japan).

### 2.6. RT-qPCR Assay

After infection, total RNA was extracted from cells using Trizol (TransGen Biotech, Beijing, China). The RNA was then reverse transcribed into cDNA using TransScript first-strand cDNA synthesis SuperMix (TransGen Biotech, Beijing, China). MonAmpTM ChemoHS Specificity Plus qPCR Mix (Monad, Shanghai, China) was utilized to measure gene expression. For the sample preparation premix, 2 μL of samples + 0.5 μL of forward primer + 0.5 μL of reverse primer + 5 μL of Premix + 2 μL of nuclease-free water were combined for a total volume of 10 μL. The RT-qPCR execution program consisted of an initial denaturation at 95 °C for 5 min followed by 35 cycles, with each cycle comprising denaturation at 95 °C for 10 s, primer annealing at 58 °C for 10 s, and extension at 72 °C for 30 s. The primer sequence used were as follows: NLRP3, F: 5′-GAGCTGGACCTCAGTGACAATGC-3′, R: 5′-ACCAATGCGAGATCCTGACAACAC-3′; ASC, F: 5′-CAGGCGAGCAGCAGCAAGAG-3′, R: 5′-CAAGAGCGTCCAGGATGGCATC-3′; Caspase1, F: 5′-ACAACCACTCGTACACGTCTTGC-3′, R: 5′-CCAGATCCTCCAGCAGCAACTTC-3′; β-actin, F: 5′-GTCAGGTCATCACTATCGGCAAT-3′, R: 5′-AGAGGTCTTTACGGATGTCAACGT-3′. Relative gene expression was determined using the 2^−ΔΔCT^ method with β-actin serving as the internal parameter.

### 2.7. ROS Assay

The Reactive Oxygen Species Assay Kit (Solarbio, CA1410, Beijing, China) was employed for the quantification of ROS levels in macrophages. DCFH-DA was diluted to a concentration of 10 μM using DMEM or RPMI1640 medium without FBS. Following *Mtb* infection, the culture medium was substituted with DCFH-DA diluent and incubated for 20 min at 37 °C under humidified conditions. Subsequently, the cells were then observed and photographed using a fluorescence microscope.

### 2.8. MDA Assay

The Malondialdehyde (MDA) Content Assay Kit (Solarbio, BC0025, Beijing, China) was utilized for the quantification of MDA in macrophages. After infection with *Mtb*, cells were harvested using an extraction solution at a ratio of 5 million cells per 1 mL of extraction solution. Cell lysis was achieved using an ultrasonic processor under the following parameters: power of 200 W; 3 s of ultraphonics; interval of 10 s; 30 repetitions. The resulting lysate was then centrifuged at 8000× *g* for 10 min to collect the supernatant. For sample preparation premixing, a total volume of 300 μL of operating fluid for MDA detection +100 μL of cell lysis supernatant +100 μL of reagent was combined in a 1.5 mL EP tube. The mixed liquid in the tube was heated at a temperature of 100 °C for 1 h. Subsequently, a volume of 200 μL of mixed liquid was transferred into 96-well plates, and the absorbances at wavelengths of both 532 nm and 600 nm were measured.

### 2.9. GSH Assay

The Reduced Glutathione (GSH) Content Assay Kit (Solarbio, BC1175, Beijing, China) was utilized for the quantification of GSH levels in macrophages. After *Mtb* infection, it was harvested using reagent one at a ratio of 5 million cells per 1 mL of extraction solution. Cell lysis was performed using an ultrasonic processor with the following parameters: power of 200 W; 3 s of ultraphonics; interval of 10 s; 30 repetitions. The lysate was then centrifuged at 12,000× *g* for 10 min to collect the supernatant as the sample. For sample preparation, a premix consisting of 20 μL of sample + 140 μL of reagent two + 40 μL of reagent three was added to a 96-well plate. After incubation at room temperature for quiescence for 2 min, absorbance at the wavelength of 412 nm was measured.

### 2.10. LDH Assay

The Lactate Dehydrogenase (LDH) Activity Assay Kit (Solarbio, BC0685, Beijing, China) was utilized for the quantification of GSH in macrophages. The cells were collected by centrifugation and resuspended with extraction solution at a ratio of 5 million cells per 1 mL of extraction solution. Cell lysis was performed using an ultrasonic processor under the following parameters: power of 200 W; ultraphonic of 3 s; interval of 10 s; 30 repetitions. After centrifugation at 8000× *g* for 10 min, the supernatant (sample) was collected. For sample preparation premixing, 10 μL of sample + 50 μL of reagent one + 10 μL of reagent two were added to a 96-well plate. The plate containing samples was incubated in a water bath at a temperature of 37 °C for a duration of 15 min. Subsequently, reagent three (50 μL) was added to the sample premix and incubated in a water bath at a temperature of 37 °C for another 15 min. Finally, reagent four (150 μL) was added to the sample premix, and quiescence was permitted at room temperature for 3 min before measuring absorbance at a wavelength of 450 nm.

### 2.11. ELISA Assay

Mouse IL-1β (MM-0040M1) and (MM-0169M1) ELISA kits were produced from mlbio (Shanghai, China). After treating the cells with SFN and *Mtb*, the supernatant of the cell culture medium was collected and centrifuged at 4000 rpm for 10 min to remove particles and polymers. The resulting supernatant was then diluted 5 times and added to an ELISA 96-well plate. Subsequently, each well was supplemented with 100 μL of HRP-conjugated reagent, covered with an adhesive strip, and incubated at 37 °C for 60 min. The plates were washed with wash solution 5 times for 1 min each time. Chromogen solution A (50 μL) and chromogen solution B (50 μL) were added to each well, gently mixed, and incubated at 37 °C for 15 min. Finally, stop solution (50 μL) was added to each well before reading the OD at a wavelength of 450 nm using a microtiter plate reader.

### 2.12. Hoechst 33342/PI Double Staining

The Hoechst 33342/PI Double Stain Kit (Solarbio, CA1120, Beijing, China) was utilized for macrophage staining. Cells were seeded in 24-well plates containing a coverslip. Following *Mtb* infection, the cells were stained in an ice bath for 30 min using a staining solution consisting of 2.5 μL of Hoechst staining solution and 2.5 μL of PI staining solution in a total volume of 500 μL. The coverslip was then washed with cold PBS and sealed with gelatin before being observed and photographed under a fluorescence microscope.

### 2.13. CCK8 Assay

The 96-well plate was seeded with approximately 50,000 primary peritoneal macrophages in each well and incubated overnight. Subsequently, SFN treatment was administered at final concentrations ranging from 0 to 100 μM in each well, including a DMSO control group. Each experimental group consisted of six replicates. After the 24 h SFN treatment, cck8 reagent was added to the cells and incubated at 37 °C for 30 m. Finally, the absorbance at 450 nm was measured using a microplate reader.

### 2.14. Statistical Analysis

Data were presented as mean ± SEM. One-way ANOVA was used to analyze the significant effects, followed by the Tukey’s HSD test (* *p* < 0.05, ** *p* < 0.01, *** *p* < 0.001). Each experiment in this study was repeated at least three times.

## 3. Results

### 3.1. The Infection of Mtb Induces Oxidative Stress in Macrophages

**Figure 1:**
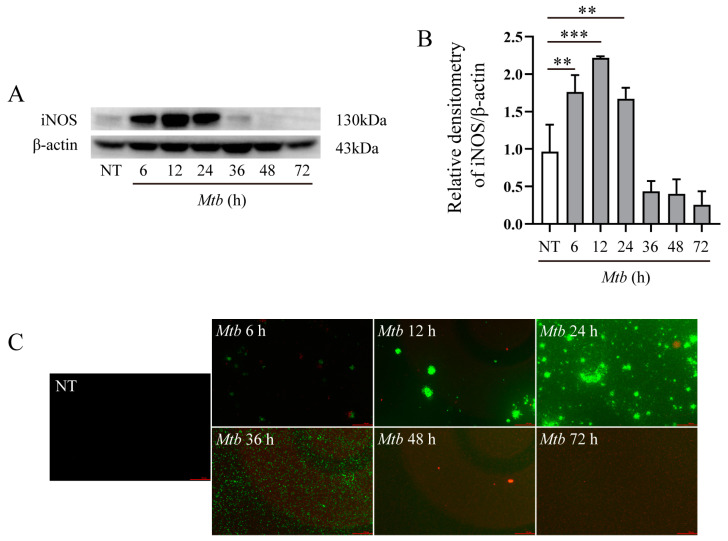
The infection of *Mtb* induces oxidative stress in macrophages. (**A**,**B**) WB analysis was performed to assess the expression of iNOS in *Mtb*-infected RAW264.7 macrophages (*n* = 3). (**C**) The Reactive Oxygen Species Assay Kit was utilized for quantifying ROS production in *Mtb*-infected RAW264.7 macrophages using a fluorescence microscope; green represents ROS, red represents *Mtb*, the images are 4×, and scale bar: 100 μm (*n* = 3). Means ± SEM, ** *p* < 0.01, and *** *p* < 0.001 represents significant difference.

To investigate the antioxidant property of SFN in *Mtb*-infected macrophages, we first examined the oxidative stress change in *Mtb*-infected macrophages. It was observed that *Mtb* infection significantly upregulated iNOS expression, particularly from 6 h to 24 h (Figure 1A,B). Moreover, *Mtb* infection notably enhanced ROS production in macrophages, especially at 24 h. These findings indicate a substantial increase in oxidative stress following *Mtb* infection, particularly at the 24 h time point. Therefore, for subsequent experiments, infected *Mtb* was utilized to induce oxidative stress over a duration of 24 h. 

### 3.2. SFN Inhibits Oxidative Stress in Mtb-Infected Macrophages by Activating NRF2 Signaling Pathway

**Figure 2:**
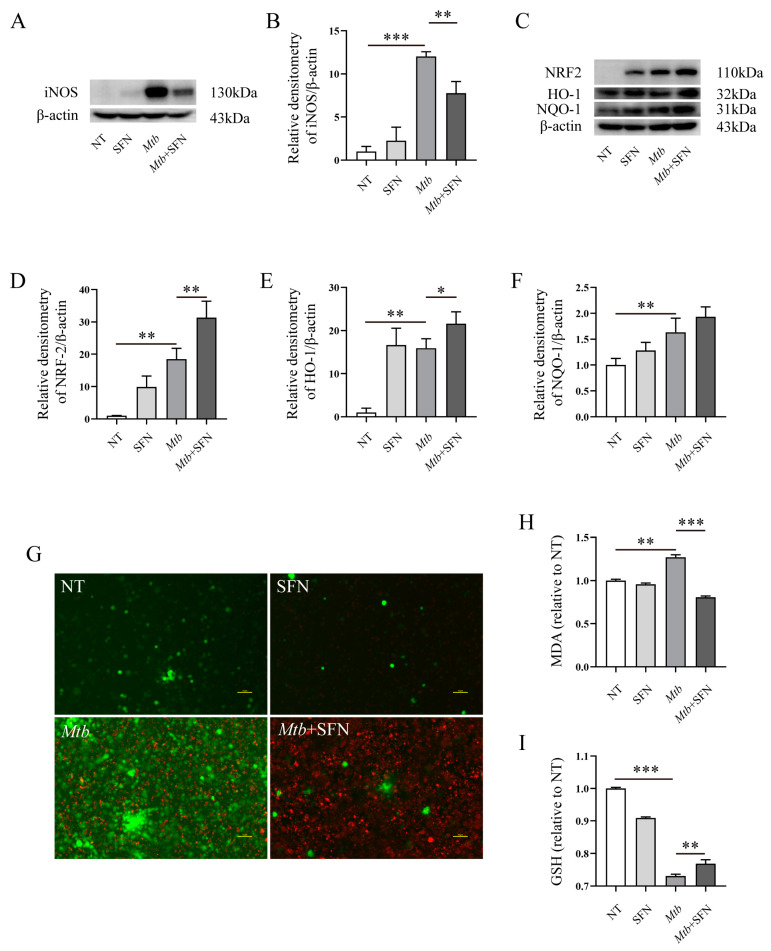
SFN inhibits oxidative stress in *Mtb*-infected RAW264.7 macrophages. Pre-treatment of the RAW264.7 macrophages with 10 μM of SFN (diluted in DMSO) for 1 h, followed by infection with *Mtb* for 24 h at an MOI value of 10. (**A**,**B**) WB analyzed the expression of iNOS in *Mtb*-infected RAW264.7 macrophages (*n* = 3); (**C**–**F**) WB analyzed the expression of NRF2, HO-1, and NQO-1 in *Mtb*-infected RAW264.7 macrophages (*n* = 3); (**G**) the Reactive Oxygen Species Assay Kit was used to measure the production of ROS in *Mtb*-infected RAW264.7 macrophages using a fluorescence microscope; green represents ROS, red represents *Mtb*, 100×, and scale bar: 10 μm (*n* = 3); (**H**,**I**) the MDA Content Assay Kit and GSH Content Assay Kit were employed to analyze the MDA and GSH content in *Mtb*-infected RAW264.7 macrophages. Means ± SEM, * *p* < 0.05, ** *p* < 0.01, and *** *p* < 0.001 represents significant difference.

We subsequently aimed to investigate the impact of SFN on oxidative stress induced by *Mtb* infection. Our findings demonstrated that supplementation with SFN effectively inhibited iNOS expression in *Mtb*-infected macrophages (Figure 2A,B), indicating its antioxidant effect during *Mtb* infection. Furthermore, we observed a significant upregulation of NRF2, HO-1, and NQO-1 upon SFN treatment (Figure 2C–F), suggesting that the antioxidant property of SFN may be attributed to the activation of the NRF2 signaling pathway. The results from ROS measurement revealed an elevation in ROS production following *Mtb* infection, which was significantly attenuated by SFN supplementation (Figure 2G). To validate these findings, we also assessed changes in MDA and GSH levels in *Mtb*-infected RAW264.7 macrophages. It was observed that *Mtb* infection increased MDA levels while it decreased GSH levels in *Mtb*-infected RAW264.7 macrophages; however, these effects were counteracted by SFN supplementation (Figure 2H,I).

**Figure 3:**
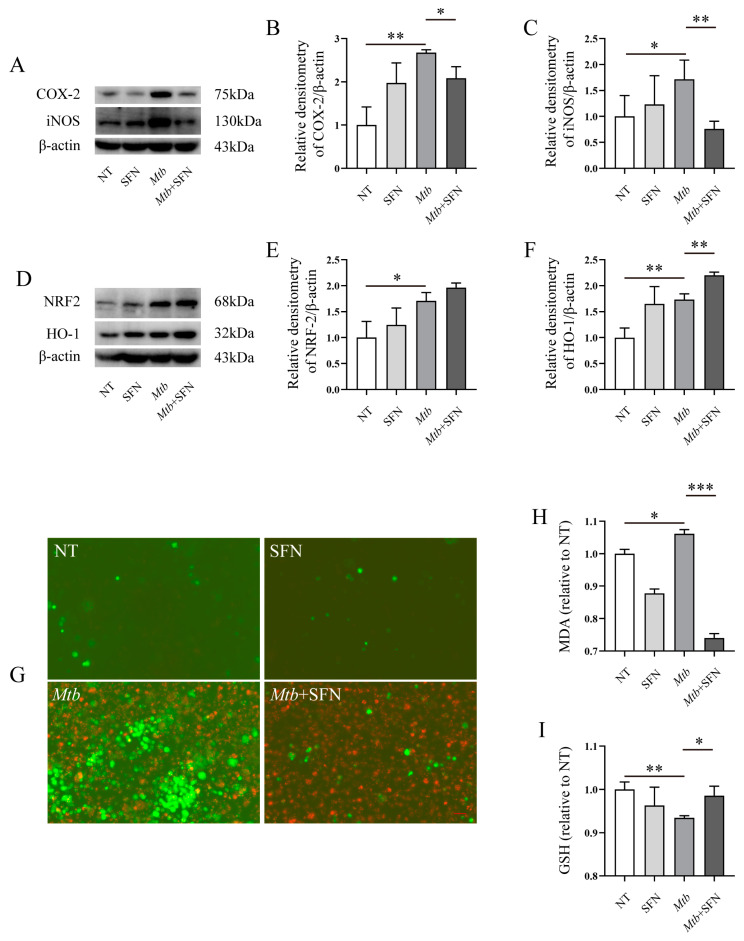
SFN inhibits oxidative stress in *Mtb*-infected primary peritoneal macrophages from WT mice. Pre-treatment of the primary peritoneal macrophages from WT mice with 10 μM of SFN (diluted in DMSO) for 1 h, followed by infection with *Mtb* for 24 h at an MOI value of 10. (**A**–**C**) WB analyzed the expression of COX-2 and iNOS in *Mtb*-infected primary peritoneal macrophages from WT mice (*n* = 3); (**D**–**F**) WB analyzed the expression of NRF2, HO-1, and NQO-1 in *Mtb*-infected primary peritoneal macrophages from WT mice (*n* = 3); (**G**) the Reactive Oxygen Species Assay Kit was used to measure the production of ROS in *Mtb*-infected primary peritoneal macrophages from WT mice; green represents ROS, red represents *Mtb*, the images are 20×, and scale bar: 100 μm (*n* = 3); (**H**,**I**) the Malondialdehyde (MDA) Content Assay Kit and Reduced Glutathione (GSH) Content Assay Kit were used to analyze the content of MDA and GSH in *Mtb*-infected primary peritoneal macrophages from WT mice. Means ± SEM, * *p* < 0.05, ** *p* < 0.01, and *** *p* < 0.001 represents significant difference.

To validate the findings, we employed *Mtb* to infect primary peritoneal macrophages from WT mice and assessed the antioxidant properties of SFN. We initially determined the optimal concentration for primary peritoneal macrophages from WT mice and observed that 50 μM and 100 μM of SFN significantly decreased cell viability, whereas concentrations ranging from 1 to 30 μM had no impact on the cells (Figure S1A). To ensure consistency with RAW264.7 cells, we also selected a concentration of 10 μM of SFN for subsequent experiments. WB results demonstrated that SFN effectively suppressed the expression of COX-2 and iNOS (Figure 3A–C). Although there was no statistically significant difference in expression between the *Mtb* and *Mtb*+SFN groups, supplementation with SFN did lead to an upregulation of NRF2 expression (Figure 3D,E). Additionally, SFN also enhanced HO-1 expression (Figure 3D,F). The immunofluorescence results demonstrated that SFN facilitated the nucleus translocation of NRF2 (Figure S2A). The ROS analysis revealed that *Mtb* infection significantly increased ROS production, while supplementation with SFN effectively reduced ROS levels (Figure 3G). Moreover, *Mtb* infection resulted in elevated MDA content and decreased GSH content; however, treatment with SFN inhibited MDA production and increased GSH content in primary peritoneal macrophages infected with *Mtb* from WT mice (Figure 3H,I). Collectively, these data indicate that SFN exerts inhibitory effects on oxidative stress in *Mtb*-infected macrophages, wherein NRF2 signaling plays a pivotal role during the infection process.

### 3.3. SFN May Inhibit the Occurrence of Pyroptosis in Mtb-Infected Macrophages

**Figure 4:**
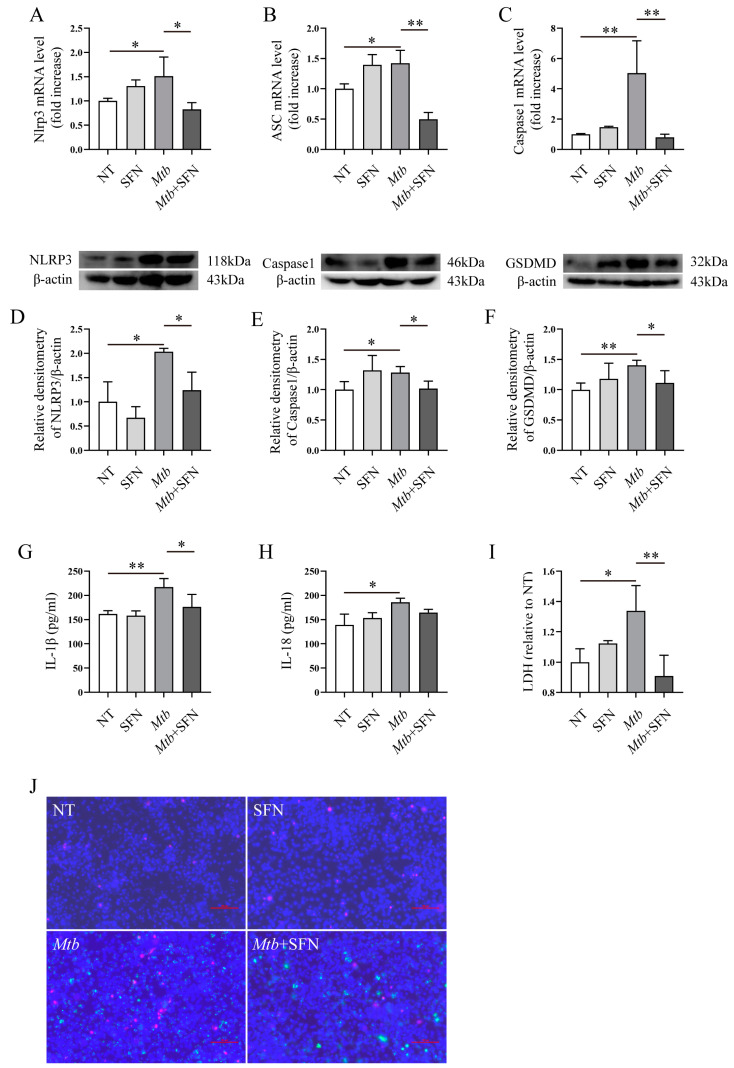
SFN may inhibit the occurrence of pyroptosis in *Mtb*-infected macrophages. Pre-treatment of the primary peritoneal macrophages from WT mice with 10 μM of SFN for 1 h, followed by infection with *Mtb* for 24 h at an MOI value of 10. (**A**–**C**) qRT-PCR analyzed the expression of NLRP3, ASC, and Caspase-1 mRNA in *Mtb*-infected primary peritoneal macrophages from WT mice (*n* = 3); (**D**–**F**) WB analyzed the expression of NLRP3, Caspase-1, and GSDMD in *Mtb*-infected primary peritoneal macrophages from WT mice (*n* = 3); (**G**,**H**) ELISA analyzed the IL-1β and IL-18 in *Mtb*-infected primary peritoneal macrophages from WT mice (*n* = 3); (**I**) the Lacate Dehydrogenase (LDH) Activity Assay Kit was used to analyze the effects of SFN on LDH in *Mtb*-infected primary peritoneal macrophages from WT mice (*n* = 3); (**J**) Hoechst 33342/PI double staining analyzed the pyroptosis in *Mtb*-infected primary peritoneal macrophages from WT mice; green represents *Mtb*, red represents PI staining, blue represents Hoechst 33,342 staining, 20×, and scale bar: 100 μm (*n* = 3); means ± SEM, * *p* < 0.05, and ** *p* < 0.01 represents significant difference.

The excessive production of ROS induces oxidative stress in macrophages. Moreover, ROS can serve as a signaling molecule to activate NLRP3 and subsequently trigger the initiation of pyroptosis in macrophages. SFN inhibits the production of ROS and oxidative stress in *Mtb*-infected macrophages. Therefore, supplementation with SFN can also inhibit pyroptosis in *Mtb*-infected macrophages. Our results demonstrated that *Mtb* infection upregulated the expression of NLRP3, ASC, and Caspase-1 mRNA, while SFN significantly downregulated their mRNA expression levels (Figure 4A–C). Further studies revealed that SFN suppressed the expression of NLRP3, Caspase-1, and GSDMD in *Mtb*-infected macrophages (Figure 4D–F), suggesting that it may possess anti-pyroptotic properties during *Mtb* infection. To further validate this finding, we utilized ELSIA to measure the levels of IL-1β and IL-18 in SFN and *Mtb* treatment macrophages. The results demonstrated that SFN effectively suppressed the expression of IL-1β induced by *Mtb* infection (Figure 4G), as well as inhibited the elevated levels of IL-18 caused by *Mtb* infection (Figure 4H). LDH is a marker of cell fragmentation and may represent pyroptosis to some extent. The results indicated that *Mtb* infection elevated LDH content within cells, whereas supplementation with SFN reduced LDH levels (Figure 4I). Hoechst 33342/PI double staining results showed that *Mtb* infection increased cell death in macrophages, whereas supplementation with SFN inhibited cell death (Figure 4J). In summary, our results demonstrated that *Mtb* infection may lead to the onset of proptosis, whereas SFN may exert an inhibitory effect in this process. 

### 3.4. SFN May Directly Inhibit Pyroptosis by Blocking the Activation of the NLRP3 Signaling Pathway

**Figure 5:**
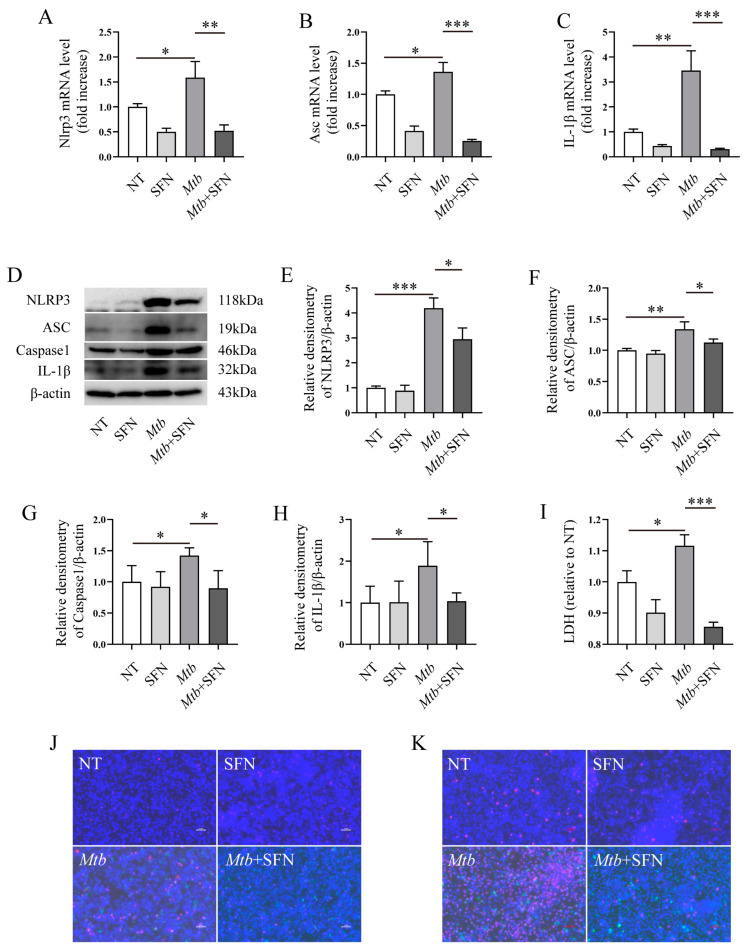
SFN may directly inhibit pyroptosis by impeding activation of the NLRP3 signaling pathway. Pre-treatment of the primary peritoneal macrophages from NRF2^−/−^ mice with 10 μM of SFN for 1 h, followed by infection with *Mtb* for 24 h at an MOI value of 10. (**A**–**C**) qRT-PCR analyzed the expression of NLRP3, ASC, and IL-1β mRNA in *Mtb*-infected primary peritoneal macrophages from NRF2^−/−^ mice (*n* = 3); (**D**–**H**) WB analyzed the expression of NLRP3, ASC, Caspase-1, and IL-1β in *Mtb*-infected primary peritoneal macrophages from NRF2^−/−^ mice (*n* = 3); (**I**) the Lacate Dehydrogenase (LDH) Activity Assay Kit was used to analyze the effects of SFN on LDH in *Mtb*-infected 24 h (**J**) (n = 3); (**J**,**K**) Hoechst 33342/PI double staining analyzed pyroptosis in *Mtb*-infected primary peritoneal macrophages from NRF2^−/−^ mice infected for 24 h (**J**) (100×, scale bar: 10 μm) and 72 h (**K**) (10×, scale bar: 100 μm) (*n* = 3). Means ± SEM, * *p* < 0.05, ** *p* < 0.01, and *** *p* < 0.001 represents significant difference.

Our findings demonstrated that SFN possesses antioxidant properties by activating the NRF2 signaling pathway and may inhibit pyroptosis during *Mtb* infection. It is noteworthy that the anti-pyroptotic property of SFN may function by activating NRF2 and reducing ROS production, thereby decreasing NLRP3-mediated pyroptosis. Subsequently, we aimed to investigate whether the potential anti-pyroptotic property of SFN depends on NRF2. Primary peritoneal macrophages were isolated from NRF2^−/−^ mice and infected with *Mtb*. The results revealed that *Mtb* infection upregulated the expression of NLRP3, ASC, and IL-1β mRNA, while supplementation with SFN inhibited their expression (Figure 5A–C). Further investigation demonstrated that SFN suppressed the expression of NLRP3, ASC, Caspase-1, and IL-1β in *Mtb*-infected primary peritoneal macrophages from NRF2^−/−^ mice (Figure 5D–H). These findings suggest that SFN may possess an anti-pyroptotic property in NRF2-deficient macrophages. Moreover, Hoechst 33342/PI double staining results indicated that SFN inhibited cell death in NRF2-deficient macrophages (Figure 5J,K). LDH results also showed that supplementation with SFN perhaps attenuated pyroptosis induced by *Mtb* infection (Figure 5I). Overall, our results indicate that SFN may exert an anti-pyroptotic property in *Mtb*-infected NRF2-deficient macrophages, suggesting a dispensable role for NRF2 in mediating the inhibitory effects of SFN on pyroptosis caused by *Mtb* infection.

## 4. Discussion

TB is a severe infectious disease caused by infection with *Mtb.* Following inhalation with *Mtb*, macrophages recognize and phagocytose the bacilli, serving as primary depots for limiting *Mtb* [28]. The invasion of *Mtb* triggers the activation of the host immune system defense mechanism by inducing macrophages to generate reactive oxygen species (ROS) [29], which can directly eradicate *Mtb* by causing oxidative damage to essential biological components such as lipids, proteins, and DNA. Due to the absence of crucial DNA repair pathways, ROS’ toxic properties lead to the demise of *Mtb* [30]. The production of ROS stimulates inflammatory and anti-microbial responses in macrophages [31] while modulating apoptosis to eliminate *Mtb* [32]. Therefore, maintaining proper oxidoreductase homeostasis is vital for the survival, persistence, and reactivation of *Mtb* [33]. To counteract ROS, *Mtb* possesses an array of protective mechanisms and enzymes including mycolic acid-rich cell wall structures, superoxide dismutase, catalase, alkyl hydroperoxidase, and peroxiredoxins that ensure its survival within macrophages [33]. The results demonstrated that the generation of ROS is advantageous for the host in eradicating *Mtb*. However, excessive ROS production beyond the body’s capacity to eliminate them subsequently leads to cellular and tissue damage, exacerbating the condition of TB patients. Moreover, several anti-mycobacterial prodrugs exhibit effectiveness only upon bioreductive activation, and oxidative stress influences the clinical outcome of TB patients [33]. Therefore, restricting ROS production and inhibiting oxidative stress could potentially serve as a method for TB treatment. In this study, we observed that *Mtb* infection induces ROS generation and oxidative stress, particularly at 24 h post-infection. The findings from RAW264.7 macrophages and primary peritoneal macrophages indicated that *Mtb* infection upregulates iNOS and COX-2 expression, whereas supplementation with SFN inhibits their expression. Further investigations revealed that SFN suppressed ROS production induced by *Mtb* infection, suggesting its ability to alleviate oxidative stress in *Mtb*-infected macrophages. To validate these results, we assessed the levels of MDA, GSH, as well as NRF2, HO-1, and NQO-1 protein expressions in *Mtb*-infected macrophages and found that SFN reduced MDA production while increasing GSH content. Additionally, SFN enhanced NRF2, HO-1, and NQO-1 expression in *Mtb*-infected macrophages. Collectively, the outcomes indicate that SFN possesses antioxidant properties during *Mtb* infection, and such properties may be attributed to the activation of the NRF2 signaling pathway.

ROS functions as a signaling molecule to activate the NRF2 signaling pathway and exerts anti-oxidant properties. Moreover, excessive ROS production induces the dissociation of thioredoxin-interacting protein (TXNIP) from thioredoxin (TRX). TXNIP plays a critical role in the assembly and activation of the NLRP3 inflammasome [34,35]. Therefore, ROS serves as an important second messenger in NLRP3 inflammasome-mediated pyroptosis [36,37]. Pyroptosis facilitates bacterial elimination; however, excessive pyroptosis leads to bacterial escape and dissemination from macrophages. SFN inhibits oxidative stress in *Mtb*-infected macrophages and may also possess an anti-pyroptotic property during *Mtb* infection. Our study demonstrated that SFN reduced the expression of NLRP3, ASC, and Caspase-1 mRNA levels as well as the expression of NLRP3, Caspase-1, and GSDMD protein levels in *Mtb*-infected macrophages, suggesting that SFN inhibited the activation of the NLRP3 signaling pathway. Further investigations revealed that SFN suppressed cell death and decreased LDH production in *Mtb*-infected macrophages. These results indicate that SFN may inhibit pyroptosis during *Mtb* infection.

**Figure 6:**
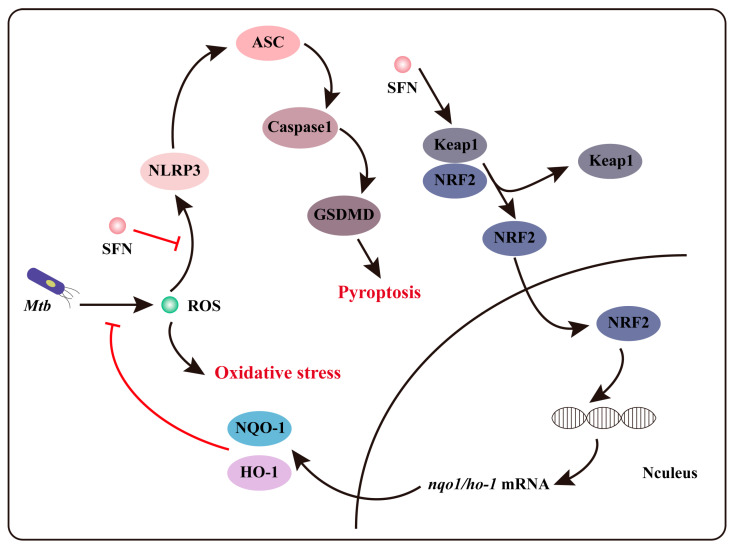
The mechanism of SFN inhibits oxidative stress and pyroptosis in *Mtb*-infected macrophages.

In our study, we discovered that SFN may exert an anti-pyroptotic property in *Mtb*-infected macrophages, and it is associated with the activation of the NRF2 signaling pathway. Previous research has demonstrated that SFN can alleviate acute gouty inflammation by inhibiting the activation of the NLRP3 inflammasome [38]. Lee et al. also reported that SFN supplementation inhibits the activation of both NLRP3 and NLRC4 inflammasomes [39], but not AIM. These findings suggest a direct inhibition of NLRP3 activation by SFN. Our study further reveals that SFN possibly exerts its anti-pyroptotic property in *Mtb*-infected macrophages by activating the NRF2 signaling pathway. However, it remains unclear whether there are alternative NRF2-independent pathways through which SFN can inhibit pyroptosis in *Mtb*-infected macrophages. To investigate whether SFN’s inhibition of pyroptosis depends on NRF2, we isolated primary peritoneal macrophages from NRF2^−/−^ mice and infected them with *Mtb*. The results showed that SFN suppressed NLRP3 expression, ROS production, and pyroptosis in *Mtb*-infected primary peritoneal macrophages from NRF2^−/−^ mice. These findings indicate that SFN may inhibit pyroptosis by activating the NRF2 signaling pathway and subsequently suppressing ROS production, while also directly suppressing NLRP3 inflammasome activation to inhibit pyroptosis in *Mtb*-infected macrophages (Figure 6).

## 5. Conclusions

In conclusion, our data demonstrate that SFN effectively inhibits oxidative stress and may exert anti-pyroptotic effects in *Mtb*-infected macrophages. The antioxidant property of SFN depends on the activation of the NRF2 signaling pathway. The excessive accumulation of ROS in the cytoplasm not only induces oxidative stress but also serves as a signal for NLRP3 inflammasome-mediated pyroptosis activation. Therefore, SFN’s potential anti-pyroptotic activity appears to be associated with NRF2 signaling pathway activation and the inhibition of ROS production. Furthermore, SFN still exerts an anti-pyroptotic property in NRF2-deficient macrophages, indicating that NRF2 is not essential to SFN’s potential anti-pyroptotic effects. In summary, our findings reveal that SFN mitigates oxidative stress through activating the NRF2 signaling pathway, while its ability to inhibit pyroptosis may be attributed to both NRF2 signaling pathway activation and NLRP3 signaling pathway inhibition in *Mtb*-infected macrophages. This study not only expands our understanding of the biological functions of SFN but also provides a new direction for screening and discovering TB drugs, which holds significant medical implications for TB treatment.

## Data Availability

Data are contained within the article.

## Supplementary Materials

The following supporting information can be downloaded at https://www.mdpi.com/article/10.3390/microorganisms12061191/s1: Figure S1: Cell viability; Figure S2: Nuclear translocation of NRF2.

## References

[1] Segal L.N., Clemente J.C., Li Y., Ruan C., Cao J., Danckers M., Morris A., Tapyrik S., Wu B.G., Diaz P. (2017). Anaerobic Bacterial Fermentation Products Increase Tuberculosis Risk in Antiretroviral-Drug-Treated HIV Patients. Cell Host Microbe.

[2] Batista L.A.F., Silva K.J.S., da Costa E.S.L.M., de Moura Y.F., Zucchi F.C.R. (2020). Tuberculosis: A granulomatous disease mediated by epigenetic factors. Tuberculosis.

[3] Suman S.K., Chandrasekaran N., Priya Doss C.G. (2023). Micro-nanoemulsion and nanoparticle-assisted drug delivery against drug-resistant tuberculosis: Recent developments. Clin. Microbiol. Rev..

[4] Houben R.M., Dodd P.J. (2016). The Global Burden of Latent Tuberculosis Infection: A Re-estimation Using Mathematical Modelling. PLoS Med..

[5] Tiemersma E.W., van der Werf M.J., Borgdorff M.W., Williams B.G., Nagelkerke N.J. (2011). Natural history of tuberculosis: Duration and fatality of untreated pulmonary tuberculosis in HIV negative patients: A systematic review. PLoS ONE.

[6] Xu G., Wang J., Gao G.F., Liu C.H. (2014). Insights into battles between Mycobacterium tuberculosis and macrophages. Protein Cell.

[7] Srivastava S., Ernst J.D., Desvignes L. (2014). Beyond macrophages: The diversity of mononuclear cells in tuberculosis. Immunol. Rev..

[8] Deramaudt T.B., Dill C., Bonay M. (2013). Regulation of oxidative stress by Nrf2 in the pathophysiology of infectious diseases. Méd. Mal. Infect..

[9] Paiva C.N., Bozza M.T. (2014). Are reactive oxygen species always detrimental to pathogens?. Antioxid. Redox Signal..

[10] Schepici G., Bramanti P., Mazzon E. (2020). Efficacy of Sulforaphane in Neurodegenerative Diseases. Int. J. Mol. Sci..

[11] Cooper D.A., Webb D.R., Peters J.C. (1997). Evaluation of the potential for olestra to affect the availability of dietary phytochemicals. J. Nutr..

[12] Winiwarter S., Bonham N.M., Ax F., Hallberg A., Lennernas H., Karlen A. (1998). Correlation of human jejunal permeability (in vivo) of drugs with experimentally and theoretically derived parameters. A multivariate data analysis approach. J. Med. Chem..

[13] Georgikou C., Yin L., Gladkich J., Xiao X., Sticht C., de la Torre C., Gretz N., Gross W., Schäfer M., Karakhanova S. (2020). Inhibition of miR30a-3p by sulforaphane enhances gap junction intercellular communication in pancreatic cancer. Cancer Lett..

[14] Lin L.C., Yeh C.T., Kuo C.C., Lee C.M., Yen G.C., Wang L.S., Wu C.-H., Yang W.-C.V., Wu A.T.H. (2012). Sulforaphane potentiates the efficacy of imatinib against chronic leukemia cancer stem cells through enhanced abrogation of Wnt/beta-catenin function. J. Agric. Food Chem..

[15] Carrasco-Pozo C., Tan K.N., Rodriguez T., Avery V.M. (2019). The Molecular Effects of Sulforaphane and Capsaicin on Metabolism upon Androgen and Tip60 Activation of Androgen Receptor. Int. J. Mol. Sci..

[16] Xu Y., Han X., Li Y., Min H., Zhao X., Zhang Y., Qi Y., Shi J., Qi S., Bao Y. (2019). Sulforaphane Mediates Glutathione Depletion via Polymeric Nanoparticles to Restore Cisplatin Chemosensitivity. ACS Nano.

[17] Ishiura Y., Ishimaru H., Watanabe T., Fujimuro M. (2019). Sulforaphane Exhibits Cytotoxic Effects against Primary Effusion Lymphoma Cells by Suppressing p38MAPK and AKT Phosphorylation. Biol. Pharm. Bull..

[18] Xie H., Chun F.K., Rutz J., Blaheta R.A. (2021). Sulforaphane Impact on Reactive Oxygen Species (ROS) in Bladder Carcinoma. Int. J. Mol. Sci..

[19] Zhang R., Miao Q.W., Zhu C.X., Zhao Y., Liu L., Yang J., An L. (2015). Sulforaphane ameliorates neurobehavioral deficits and protects the brain from amyloid beta deposits and peroxidation in mice with Alzheimer-like lesions. Am. J. Alzheimer’s Dis. Other Dement..

[20] Morroni F., Sita G., Djemil A., D’amico M., Pruccoli L., Cantelli-Forti G., Hrelia P., Tarozzi A. (2018). Comparison of Adaptive Neuroprotective Mechanisms of Sulforaphane and its Interconversion Product Erucin in in Vitro and in Vivo Models of Parkinson’s Disease. J. Agric. Food Chem..

[21] Pu Y., Qu Y., Chang L., Wang S.M., Zhang K., Ushida Y., Suganuma H., Hashimoto K. (2019). Dietary intake of glucoraphanin prevents the reduction of dopamine transporter in the mouse striatum after repeated administration of MPTP. Neuropsychopharmacol. Rep..

[22] Li B., Cui W., Liu J., Li R., Liu Q., Xie X.-H., Ge X.-L., Zhang J., Song X.-J., Wang Y. (2013). Sulforaphane ameliorates the development of experimental autoimmune encephalomyelitis by antagonizing oxidative stress and Th17-related inflammation in mice. Exp. Neurol..

[23] Choi K.-M., Lee Y.-S., Kim W., Kim S.J., Shin K.-O., Yu J.-Y., Lee M.K., Lee Y.-M., Hong J.T., Yun Y.-P. (2014). Sulforaphane attenuates obesity by inhibiting adipogenesis and activating the AMPK pathway in obese mice. J. Nutr. Biochem..

[24] Yao A., Shen Y., Wang A., Chen S., Zhang H., Chen F., Chen Z., Wei H., Zou Z., Shan Y. (2015). Sulforaphane induces apoptosis in adipocytes via Akt/p70s6k1/Bad inhibition and ERK activation. Biochem. Biophys. Res. Commun..

[25] Martin F., van Deursen J.M., Shivdasani R.A., Jackson C.W., Troutman A.G., Ney P.A. (1998). Erythroid maturation and globin gene expression in mice with combined deficiency of NF-E2 and nrf-2. Blood.

[26] Wakabayashi N., Itoh K., Wakabayashi J., Motohashi H., Noda S., Takahashi S., Imakado S., Kotsuji T., Otsuka F., Roop D.R. (2003). Keap1-null mutation leads to postnatal lethality due to constitutive Nrf2 activation. Nat. Genet..

[27] Fuse Y., Kobayashi M. (2017). Conservation of the Keap1-Nrf2 System: An Evolutionary Journey through Stressful Space and Time. Molecules.

[28] Pieters J. (2008). Mycobacterium tuberculosis and the macrophage: Maintaining a balance. Cell Host Microbe.

[29] Smith I. (2003). Mycobacterium tuberculosis pathogenesis and molecular determinants of virulence. Clin. Microbiol. Rev..

[30] Kurthkoti K., Varshney U. (2012). Distinct mechanisms of DNA repair in mycobacteria and their implications in attenuation of the pathogen growth. Mech. Ageing Dev..

[31] Bhat S.A., Singh N., Trivedi A., Kansal P., Gupta P., Kumar A. (2012). The mechanism of redox sensing in Mycobacterium tuberculosis. Free Radic. Biol. Med..

[32] Behar S.M., Martin C.J., Booty M.G., Nishimura T., Zhao X., Gan H.-X., Divangahi M., Remold H.G. (2011). Apoptosis is an innate defense function of macrophages against Mycobacterium tuberculosis. Mucosal Immunol..

[33] Kumar A., Farhana A., Guidry L., Saini V., Hondalus M., Steyn A.J. (2011). Redox homeostasis in mycobacteria: The key to tuberculosis control?. Expert Rev. Mol. Med..

[34] Zhou R., Tardivel A., Thorens B., Choi I., Tschopp J. (2010). Thioredoxin-interacting protein links oxidative stress to inflammasome activation. Nat. Immunol..

[35] Han Y., Xu X., Tang C., Gao P., Chen X., Xiong X., Yang M., Yang S., Zhu X., Yuan S. (2018). Reactive oxygen species promote tubular injury in diabetic nephropathy: The role of the mitochondrial ros-txnip-nlrp3 biological axis. Redox Biol..

[36] Yu X., Lan P., Hou X., Han Q., Lu N., Li T., Jiao C., Zhang J., Zhang C., Tian Z. (2017). HBV inhibits LPS-induced NLRP3 inflammasome activation and IL-1β production via suppressing the NF-κB pathway and ROS production. J. Hepatol..

[37] Muñoz-Planillo R., Kuffa P., Martínez-Colón G., Smith B.L., Rajendiran T.M., Núñez G. (2013). K⁺ efflux is the common trigger of NLRP3 inflammasome activation by bacterial toxins and particulate matter. Immunity.

[38] Yang G., Yeon S.H., Lee H.E., Kang H.C., Cho Y.Y., Lee H.S., Lee J.Y. (2018). Suppression of NLRP3 inflammasome by oral treatment with sulforaphane alleviates acute gouty inflammation. Rheumatology.

[39] Lee J., Ahn H., Hong E.J., An B.S., Jeung E.B., Lee G.S. (2016). Sulforaphane attenuates activation of NLRP3 and NLRC4 inflammasomes but not AIM2 inflammasome. Cell. Immunol..

