# Supplementary material for: Sulforaphane Inhibits Oxidative Stress and May Exert Anti-Pyroptotic Effects by Modulating NRF2/NLRP3 Signaling Pathway in Mycobacterium tuberculosis-Infected Macrophages
Source: Microorganisms. 2024 Jun 13;12(6):1191. doi: 10.3390/microorganisms12061191 (PMC11205970; doi:10.3390/microorganisms12061191)
Supplement: Supplementary file 1 [file microorganisms-12-01191-s001.zip › microorganisms-2985313-supplementary.pdf]

Supplementary figures

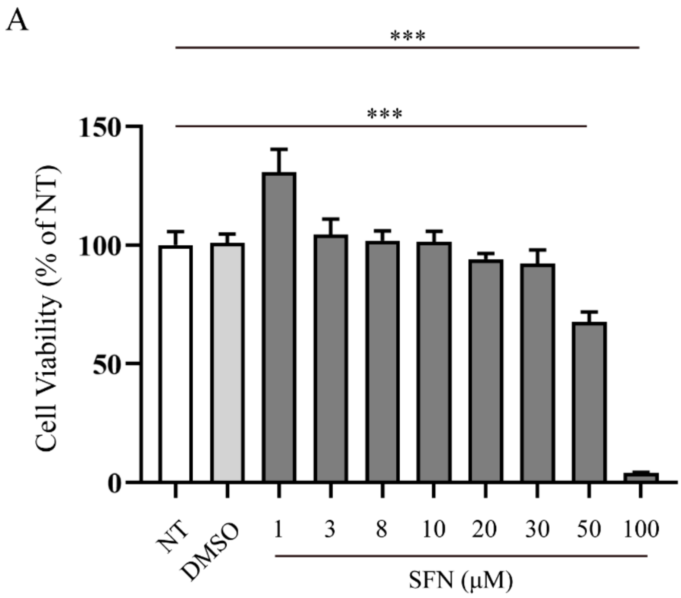

Figure S1. Cell viability. (**A**) The effects of various concentrations of SFN on primary peritoneal macrophages from WT mice ($n = 6$).

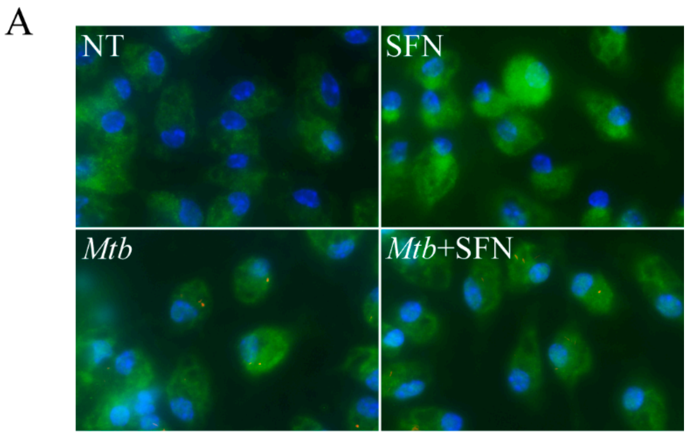

Figure S2. Nuclear translocation of NRF2. (**A**) Nuclear translocation of NRF2 in Mtb-infected primary peritoneal macrophages from WT mice, Green representative NRF2, Red representative Mtb, Blue representative nucleus, the images are 100 ×, scale bar: 200 μm ($n = 3$).
